# A subset of dendritic cells as a major and constitutive source of IL-22BP

**DOI:** 10.1186/1479-5876-9-S2-P2

**Published:** 2011-11-23

**Authors:** Jérôme Martin, Gaelle Bériou, Michèle Heslan, Céline Bossard, Anne-Chantal Knol, Brigitte Dréno, Simon Milling, Miriam Merad, Régis Josien

**Affiliations:** 1 INSERM U643, ITUN, Nantes, France; 2 Laboratoire d’Immunologie, Nantes, France; 3 Service d’Anatomo-Pathologie, Nantes, France; 4 INSERM U892, Laboratoire d’Immuno-Dermatologie, CHU Nantes, Nantes, France; 5 University of Glasgow, Glasgow, UK; 6 Mount Sinai School of Medicine, New York, USA

## Introduction

IL-22 is a cytokine produced by T cells and innate lymphocytes. Its receptor is exclusively expressed by non hematopoietic cells mostly epithelial cells and hepatocytes. IL-22 has pathogenic or protective effects depending on the model. A role for IL-22 is suggested in IMID such as psoriasis, rheumatoid arthritis and Crohn disease. IL-22BP is a soluble inhibitory receptor specific for IL-22 whose cellular source and physiological role are mainly unknown.

## Aims

To identify the cellular source of IL-22BP in vivo and its regulation of expression.

## Methods

Rat dendritic cells (DCs) were prepared from spleen. Human DCs were derived from monocytes (MDDC) in the presence of GM-CSF+IL-4. IL-22BP mRNA expression was assessed by q-PCR. Immunostaining experiments were performed with polyclonal and/or monoclonal Ab to IL-22BP. IL-22BP in serum was identified by WB.

## Results

IL-22BP mRNA was expressed at high levels in rat secondary lymphoid organs and intestine. In spleen, IL-22BP mRNA was restricted to a CD4^+^SIRPA^+^ subset of DCs. This expression was constitutive. Similar results were obtained in lymph nodes. IL-22BP expression in DCs was confirmed at the protein level. An even stronger expression was found in a subset of lymph DCs migrating from gut. In mouse, IL-22BP expression appeared restricted to the CD103^+^ subsets of intestine DCs. In human, immunostaining experiments identified stellate IL-22BP^+^ cells in colon lamina propria and dermis. In vitro, IL-22BP expression was induced during MDDC differentiation and retinoic acid receptor alpha agonist dramatically enhanced this expression. IL-22BP expression was rapidly down-regulated following maturation both in rat and human DC. Finally, WB analysis revealed high levels of serum IL-22BP in healthy volunteers.

## Conclusion

These results indicate that tissue and lymphoid DCs are a main source of IL-22BP suggesting a role for these DCs in regulating IL-22 at epithelial barriers.

